# Acceptance of HPV Vaccination: A Systematic Review of Knowledge, Attitudes and Barriers Among Healthcare Practitioners in Low‐ and Middle‐Income Countries

**DOI:** 10.1155/bmri/9294978

**Published:** 2026-01-16

**Authors:** Nancy Innocentia Ebu Enyan, Mengying Zhang, Sebastian Ken-Amoah, Michelle King-Okoye, Joyce Agyeiwaa, Richard Sarfo-Walters, Dorothy Addo-Mensah, Patrick Kafui Akakpo, Dorcas Obiri-Yeboah, Lawrence Doi

**Affiliations:** ^1^ Department of Public Health Nursing, University of Cape Coast, Cape Coast, Ghana, ucc.edu.gh; ^2^ Nursing Studies, School of Health in Social Science, The University of Edinburgh, Edinburgh, UK, ed.ac.uk; ^3^ Scottish Collaboration for Public Health Research and Policy, The University of Edinburgh, Edinburgh, UK, ed.ac.uk; ^4^ Department of Obstetrics and Gynaecology, University of Cape Coast, Cape Coast, Ghana, ucc.edu.gh; ^5^ Rosemary Bryant Research Centre, University of South Australia, Adelaide, Australia, unisa.edu.au; ^6^ Department of Maternal and Child Health, University of Cape Coast, Cape Coast, Ghana, ucc.edu.gh; ^7^ Department of Adult Health, University of Cape Coast, Cape Coast, Ghana, ucc.edu.gh; ^8^ Department of Anatomic Pathology, University of Cape Coast, Cape Coast, Ghana, ucc.edu.gh; ^9^ Department of Microbiology and Immunology, University of Cape Coast, Cape Coast, Ghana, ucc.edu.gh

**Keywords:** attitudes, healthcare practitioners, HPV vaccination acceptance, low- and middle-income countries

## Abstract

**Background:**

Cervical cancer is one of the diseases that reflects global inequities. Vaccination against the human papillomavirus (HPV) is one of the main pillars of the World Health Organization (WHO) 2030 cervical cancer 90:70:90 elimination strategy. The role of healthcare practitioners in HPV vaccination acceptance cannot be overemphasized. This review investigated healthcare practitioners′ knowledge, attitudes and barriers to promoting HPV vaccination in low‐ and middle‐income countries (LMICs).

**Methods:**

A comprehensive search for relevant literature from 2006 to 2024 was conducted in the following databases: Web of Science, Scopus, MEDLINE, EMBASE, PsycINFO (via Ovid), Cochrane Library and CINAHL PLUS (via EBSCOhost). The included studies were published in the English language. Screening of eligible studies, data extraction and quality assessment were conducted in duplicate. The data was synthesise using narrative synthesis.

**Results:**

A total of 671 papers were identified from the database search, with seven studies meeting the criteria for inclusion. This review demonstrates varied levels of awareness, knowledge and attitudes of 136 healthcare practitioners in LMICs towards HPV vaccination. Although some studies demonstrated a positive attitude, others reported resistance towards the vaccine. The perceived barriers to HPV vaccination by healthcare practitioners identified were interpersonal, community‐level and systemic in nature. Additionally, acceptance of HPV vaccination varied across the studies.

**Conclusions:**

This review highlights the need for capacity building programmes for healthcare practitioners in the area of HPV vaccination to enhance their knowledge and attitudes and develop contextually relevant interventions to eliminate the many barriers they encounter.

## 1. Background

Cervical cancer poses a major global health challenge. It ranks as the fourth most common cancer in women worldwide [1, 2]. Despite being a preventable disease, cervical cancer remains a major cause of cancer‐related deaths in women. It is estimated that in 2022, cervical cancer was diagnosed in about 660,000 women, with a resultant 350,000 deaths globally [3].

Few diseases reflect global inequities as much as cervical cancer, with disproportionate disease burden and death occurring in low‐ and middle‐income countries (LMICs), where access to preventive healthcare services, including screening and vaccination, is often limited or non‐existent [4–6]. In 2022, about 80% of the 660,000 new cases of cervical cancer and 90% of the 350,000 related deaths occurred in LMICs [2, 7, 8].

Cervical cancer is caused almost exclusively by persistent infection with high‐risk genotypes of the human papillomavirus (HPV), a common virus transmitted mainly through sexual contact [3]. HPV is also associated with other cancers, but cervical cancer is, by far, the most common HPV‐related cancer, accounting for over 90% of all HPV‐related cancers [1, 9, 10].

HPV vaccine plays a vital role in cervical cancer prevention; it protects against the most common oncogenic types of the virus. Its introduction has proven to be an effective preventive strategy in reducing the incidence of cervical cancer and other HPV‐related diseases [11, 12]. For instance, evidence from England suggests an 87% reduction in cervical cancer rates in women vaccinated at ages 12–13, whereas those vaccinated at ages 14–16 and 16–18 saw reductions of 62% and 34%, respectively, compared with unvaccinated women [13].

Recognizing its importance, the World Health Organization (WHO) incorporated HPV vaccination in its global strategy to eliminate cervical cancer as a public health problem by 2030, aiming to achieve 90% HPV vaccine coverage among girls by age 15 [3, 14]. However, progress towards this target has been uneven within the global community, with LMICs facing significant challenges in achieving the target [15]. Among barriers attributed to this disparity are limited availability of vaccines, cultural resistance, misinformation, disinformation and gaps in healthcare practitioners′ (HCPs) knowledge and understanding of HPV and its vaccines [16–19].

HCPs are uniquely positioned to surmount these challenges and promote HPV vaccine uptake. As trusted sources of health information, HCPs play a vital role in educating communities and recommending vaccination, which is known to be strongly associated with higher HPV vaccination rates [20]. However, evidence suggests that HCPs often fail to provide the necessary health education about the vaccine, contributing to its low uptake [21, 22].

Several factors contribute to HCPs′ inadequate engagement in promoting HPV vaccination. Gaps in knowledge and misinformation about HPV and the vaccine′s safety and efficacy are common [23, 24]. Negative attitudes, including concerns about vaccine safety, further hinder their ability to advocate for vaccination [25, 26]. Beyond knowledge gaps and attitudes, systemic and cultural barriers in LMICs, such as inadequate healthcare infrastructure, unavailability of vaccines, high costs and societal stigma surrounding sexual health, complicate their efforts [16, 27].

Addressing these challenges requires a comprehensive understanding of HCPs′ knowledge, attitudes and barriers to promoting HPV vaccination, particularly in LMICs, where the need for effective cervical cancer prevention strategies is most urgent. Although there have been studies that explored these factors in individual LMICs, there is a lack of a comprehensive, systematic review that integrates findings across multiple countries. This gap in the literature poses a challenge in developing evidence‐based strategies to improve HPV vaccine uptake among healthcare workers and, by extension, the general population.

To address this, we conducted a systematic review of existing literature on HCPs′ knowledge, attitudes and barriers relating to HPV vaccination in LMICs, with the purpose of providing a deeper understanding of their challenges in promoting HPV vaccine uptake. The findings will inform the development of tailored public health strategies and policy interventions to enhance HPV vaccine acceptance among HCPs and, ultimately, contribute to global efforts to eliminate cervical cancer. The following questions guide this review:

1. What is the knowledge of HCPs in LMICs regarding HPV vaccination?
2. What are the attitudes of HCPs in LMICs towards HPV vaccination?
3. What are the perceived barriers to HPV vaccination among HCPs in LMICs?
4. To what extent do HCPs in LMICs accept HPV vaccination?

## 2. Methods

### 2.1. Design

A qualitative systematic review and evidence synthesis was conducted using the Preferred Reporting Items for Systematic Reviews and Meta‐Analysis (PRISMA) statement [28]. The review protocol was prospectively registered in Prospero on 17 October 2024 with Protocol Registration Number CRD42024598195.

### 2.2. Search Strategy and Eligibility Criteria

A comprehensive search was conducted in the following databases: Web of Science, Scopus, MEDLINE, EMBASE, PsycINFO (via Ovid), Cochrane Library and CINAHL PLUS (via EBSCOhost). The search strategy was adapted for the different databases to maximize sensitivity and specificity. Boolean operators were employed to guide the search. Table 1 shows the search strategy, using the population, phenomenon of interest, context and outcome (PICO) framework.

**Table 1:** Search terms using PICO framework.

Population	‘health∗ practitioner∗’ or ‘health care practitioner∗’ or ‘general practitioner∗’ or nurse∗ or pharmacist∗ or midwi∗ or doctor∗ or ‘health∗ student∗’ or ‘health care student∗’ or ‘nursing student∗’ or clinician∗
Intervention	Cervarax or Gardasil or ‘HPV Vaccin∗’ or ‘Papillomavirus Vaccin∗’ or ‘Human Papillomavirus Recombinant Vaccine Quadrivalent’ or ‘human papilloma virus vaccin∗’
Context	lmic∗ or ‘low income countr∗’ or ‘low income nation∗’ or ‘middle income countr∗’ or ‘middle income nation∗’ or ‘developing countr∗’ or ‘developing nation∗’ or ‘less developed countr∗’ or ‘less developed nation∗’ or ‘least developed countr∗’ or ‘least developed nation∗’ or ‘third‐world nation∗’ or ‘third‐world nation∗’ or ‘third‐world countr∗’ or ‘third‐world countr∗’ or ‘underdeveloped countr∗’ or ‘under developed nation∗’ or ‘poor countr∗’ or HINARI or Africa or ‘Southeast Asia’ or China or India or Afghanistan or ‘Burkina Faso’ or Burundi or ‘Central African Republic’ or Chad or Congo or Eritrea or Ethiopia or Gambia or ‘Guinea‐Bissau’ or Korea or Liberia or Madagascar or Malawi or Mali or Mozambique or Niger or Rwanda or ‘Sierra Leone’ or Somalia or Sudan or Syria∗ or Togo or Uganda or Yemen or Angola or Bangladesh or Benin or Bhutan or Bolivia or ‘Cabo Verde’ or Cambodia or Cameroon or Comoros or ‘Cote d′Ivoire’ or Djibouti or Egypt or Eswatini or Ghana or Guinea or Haiti or Honduras or India or Jordan or Kenya or Kiribati or Kyrgyz or Lao or Lebanon or Lesotho or Mauritania or Micronesia or Morocco or Myanmar or Nepal or Nicaragua or Nigeria or Pakistan or Papua or Philippine∗ or Samoa or ‘Sao Tome’ or Principe or Senegal or ‘Solomon Islands’ or ‘Sri Lanka’ or Tajikistan or Tanzania or ‘Timor‐Leste’ or Tunisia or Uzbekistan or Vanuatu or Vietnam or ‘West Bank’ or Gaza or Zambia or Zimbabwe
Outcome	perspect∗ or belief∗ or attitude∗ or view∗ or opinion∗ or experien∗ or barrier∗ or knowledge or accept∗ or facilitat∗ or perception∗

We carried out the literature search from January 2006 up to 30 September 2024. This timeframe was chosen, because HPV vaccine was introduced in June 2006. We focused on studies published in English in LMICs. Additional inclusion and exclusion criteria are detailed in Table 2. To pictorially represent the study selection process, we adapted the PRISMA flow diagram [29].

**Table 2:** Inclusion and exclusion criteria.

**Domain**	**Inclusion**	**Exclusion**
Population (P)	HCPs, including nurses, pharmacists, midwives, disease control experts, doctors and healthcare students	Patients and nonhealthcare students
Phenomenon of Interest (I)	HPV vaccination	Papers that do not focus on HPV vaccination
Context (Co)	Low‐ and middle‐income countries	High‐income countries
Outcome (O)	Knowledge, attitude or barriers	Focus not on knowledge, attitude or barriers
Study type	Primary studies	Reviews
Year	January 2006 to September 2024	Studies published before January 2006
Language	English	Other languages

### 2.3. Data Management and Selection Process

**Figure 1:**
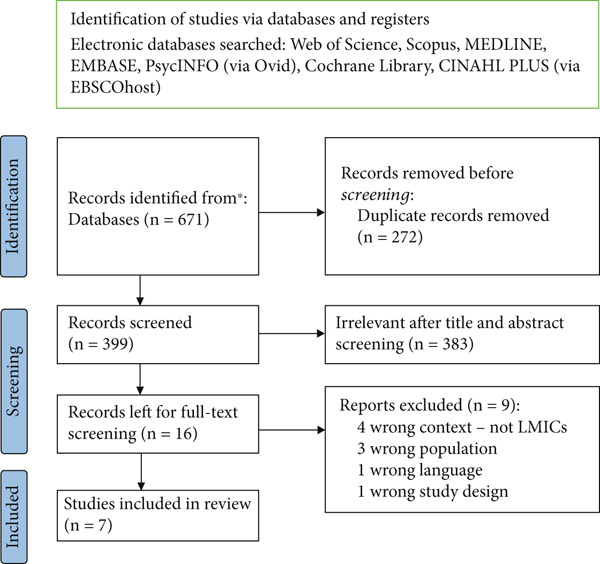
PRISMA flow diagram illustrating a systematic study selection process.

The data management process was conducted using Covidence [30]. Four authors (M.Z., L.D., D.A‐M. and M.K‐O.) independently screened all the potentially relevant papers in a two‐phase process. During the first phase, titles and abstracts were reviewed to assess for possible inclusion. In the second phase, full‐text articles were evaluated for relevance and eligibility criteria were applied to identify the most suitable studies for inclusion. Each reviewer independently documented the reasons for exclusions. These reasons are captured in Figure 1. Any conflicts or discrepancies were resolved through regular discussions with all authors. Figure 1 presents a PRISMA flow diagram illustrating the systematic study selection process.

### 2.4. Data Extraction

Relevant data from the included studies that address the review questions were extracted in a well‐structured format to facilitate synthesis and interpretation of findings. To ensure accuracy and consistency, two authors (M.K‐O. and R.S‐W.) independently conducted the data extraction and any discrepancies that arose were discussed and resolved. Microsoft Excel was used to organize the extracted data, which included key details such as author(s), year and country of study, purpose of study, study design, study population, sample, knowledge of HPV vaccination, barriers to HPV vaccination, attitude towards HPV vaccination and acceptance rate of HPV vaccination. The data extraction process adhered to the PRISMA guidelines to ensure rigour. See Table 3 for the characteristics of the included studies.

**Table 3:** The characteristics of the included studies.

**Author/year/country**	**Purpose**	**Sample/participants and recruitment**	**Data collection and analysis**	**Knowledge of HPV vaccination**	**Barriers to HPV vaccination**	**Attitudes to HPV vaccination**	**Acceptance of HPV vaccination**
Balogun and Omotade (2022) Nigeria [31]	To explore facilitators and barriers for the recommendation of HPV vaccine for adolescents by healthcare workers in Ibadan, Nigeria.	14 healthcare workers (eight nurses, four doctors, one medical social worker and one health visitor). Recruited from two primary health centres and one secondary health centre	Key informant interviews conducted by two medical students, analysed with content analysis, following the philosophy of theoretical domains′ framework.	Almost all the healthcare workers had knowledge of the HPV vaccine. Females had more detailed knowledge about the different types of HPV vaccines, as well as their doses. Some nurses from the primary health centres had little or no knowledge about the vaccine.	Some traditional beliefs about cancers were identified as barriers for the recommendation of the vaccine. Other barriers in the environmental context and resources include vague government policies about HPV vaccine for adolescents and the high cost of the vaccine.	Most of the healthcare workers believed that it was their responsibility to recommend HPV vaccine for adolescents and motivate them. Most believed they could recommend the vaccine for adolescents, with exception of one nurse due to lack of required knowledge.	Optimism about the recommendation of the vaccine was seen in the responses of only two healthcare workers, and they believed that their continued recommendation of the vaccine will eventually lead to the acceptance of the vaccine for adolescents.

Crann et al. (2016) Rural Zimbabwe [32]	To examine healthcare providers′ perceptions of current hospital practices and issues in cervical cancer prevention and treatment and knowledge of HPV and HPV vaccines and perspectives on introducing HPV vaccination programmes including potential facilitators and barriers to successful implementation.	15 healthcare providers (nine nurses, two doctors, two counsellors, one educator and one midwife). Recruited from a mission hospital	Semistructured interviews. Thematic analysis	Very limited knowledge and lack of awareness of HPV and HPV vaccine; many participants were unaware of HPV′s transmission and its link to cervical cancer.	Identified sociocultural challenges (concerns around sexual activity, adolescents and parental consent and logistical infrastructure for safe vaccine delivery (cost and schedule; lack of refrigeration and unreliable electricity).	Optimistic and supportive of HPV vaccination, recognizing its potential and effectiveness to reduce cervical cancer. Less optimistic of successful implementation	Cultural stigmas around acceptance of HPV vaccine

Kataria et al. (2022) West Bengal, India [33]	To understand awareness, perceptions and choices of physicians when recommending the HPV vaccine to parents of adolescent girls.	32 physicians (16 paediatricians, five gynaecologists, five gynaecologic oncologists, four general physicians and two public health specialists), (nearly half in private practice). Recruited from public and private practice	In‐depth interviews analysed using thematic analysis	High knowledge; all physicians were aware of HPV and its vaccine, though knowledge varied by specialty.	Parental hesitancy towards the HPV vaccines, including cost and lack of national guidelines in India, including practice‐level barriers.	Attitudes were generally favourable but some physicians were hesitant due to parental concerns and cultural barriers.	There were varied experiences related to acceptance of HPV vaccines. Recommendations were often not routine, which were influenced by various factors.

Krupp et al. (2010) Mysore, India [34]	To investigate physician intention to recommend the HPV vaccine to parents of adolescent girls in India.	20 physicians (nine paediatrics, six obstetrics/gynaecology and five family/general practice). Recruited from Mysore district.	Semistructured qualitative interviews. Iterative analytic process.	Limited knowledge and basic awareness of HPV‐cervical cancer connection.	Highlighted that cost and cultural stigma and sensitivity about discussing sexuality. HPV vaccines were not part of the universal immunization programme.	Attitudes were positive overall to HPV vaccine. However, hesitancy was noted when recommending HPV vaccine due to knowledge gaps and barriers.	Acceptance of HPV vaccines were limited; most patients are unlikely to accept recommendations.

Shetty et al. (2021) Mangaluru, India [35]	To evaluate undergraduate students′ understanding towards barriers and facilitators of HPV vaccination and identify potential health education opportunities for HPV vaccination.	20 undergraduate healthcare students (11 males and nine females) across medicine, dentistry, nursing and medical lab programmes. Age 18–26 years, recruited from one medical college.	Two focus group discussions (2017) analysed with thematic content analysis.	High knowledge of HPV infection, including its risks and signs and symptoms, moderate knowledge; increased awareness of HPV vaccines among female students compared with male students. Nursing and dental students had less knowledge than medical students	Unsure about safety concerns. Cultural barriers related to people′s perceptions. Inaccessible and not cost‐effective	Male students —hesitant attitudes towards HPV vaccination, female positive. Males are embarrassed but some females were optimistic towards receiving HPV vaccination.	Acceptance of HPV vaccines were higher among female students compared with male students

Venturas and Umeh (2017) Zambia [36]	To explore health professionals′ perspectives on the HPV vaccination programme in Zambia.	15 healthcare professionals (12 nurses, two gynaecologists and one oncologist) across government, private and missionary hospitals. Recruited from several health organisations in Kabwe, and also a private hospital/clinic, government hospital and district health centre in Lusaka	Semistructured interviews analysed with thematic analysis.	Knowledge varied across HCPs, those in urban areas were more informed than in rural, but misconceptions expressed.	Economic concerns and cultural myths were highlighted including lack of resources in rural areas.	There were positive attitudes towards the HPV vaccine among educated professionals, but fears and myths reduced confidence in vaccine advocacy.	Acceptance of HPV vaccine was limited; there are myths that exist towards accepting the HPV vaccine.

Wong (2011) Malaysia [37]	To investigate physician′s practice behaviour, attitudes and issues surrounding HPV recommendation. Secondly, the study sought to determine the promoters and inhibitors of accepting HPV vaccination in multiculture and multireligion communities.	20 physicians (13 family physicians, four gynaecologists and three paediatricians). Eight Chinese, seven Indians and five Malays.	In‐depth qualitative interviews using a semistructured guide. Grounded theory methodology	Most physicians were aware of HPV vaccines but expressed concerns about HPV vaccines long‐term efficacy and protection.	The high cost of HPV vaccines and cultural misconceptions such as promoting sexual activity. There was limited public awareness highlighted.	Happy to prescribe and recommend the HPV vaccine, but barriers such as cost and parental concerns influenced these. Gynaecologists were always more likely to recommend compared with other HCPs.	Many obstacles hinder HPV vaccine acceptance.

### 2.5. Quality Assessment

The methodological rigour and overall quality of each of the included studies were assessed by two authors (M.K‐O. and R.S‐W.) using the Joanna Briggs Institute (JBI) critical appraisal checklist for qualitative research. With the JBI appraisal tool, the risk of bias in individual studies was assessed by examining key domains, such as clarity of research objectives, appropriateness of the study design, data collection methods and the robustness of data analysis. The scores for the critical appraisal of the included studies were 10/10, 9/10, 9/10, 8/10, 8/10, 7/10 and 7/10 as indicated in File S1.

### 2.6. Data Analysis

The Economic and Social Research Council′s narrative synthesis framework served as the foundation for the narrative synthesis process [38]. This approach facilitated a structured synthesis of the included studies. Initially, a preliminary analysis was conducted involving the tabulation of findings of the studies and grouping them based on the review questions. This step ensured a clear alignment between the data extracted and the objectives of the review. The characteristics of the studies, their reported findings and the relationships within and across studies were carefully examined to identify patterns, differences and similarities. This meticulous examination highlighted key themes that emerged from the data. To ensure rigour and credibility of the results, the robustness of the synthesis was evaluated by examining the consistency and coherence of the findings and assessing the potential for bias across the included studies.

### 2.7. Patient and Public Involvement

It was not appropriate or possible to involve patients or the public in the design, conduct, reporting or dissemination plans of our research.

## 3. Results

A total of 671 papers were identified through the database search, with seven papers meeting the criteria for inclusion in this review. The process of identifying, screening and selecting papers is illustrated in Figure 1; it outlines the flow of records from identification to final inclusion in the review.

The included papers published between 2010 and 2022 explored the perspectives of 136 HCPs across five LMICs: India (*n* = 3), Nigeria (*n* = 1), Zimbabwe (*n* = 1), Malaysia (*n* = 1) and Zambia (*n* = 1). They reported on their awareness and knowledge, attitudes, barriers and acceptance towards HPV vaccination. Thematic domains and their corresponding evidence sources are provided in Table 4.

**Table 4:** Thematic domains and their corresponding evidence sources.

**Themes**	**Evidence sources**							
	**Balogun and Omotade (2022) [** **31** **]**	**Crann et al. (2016) [** **32** **]**	**Kataria et al. (2022) [** **33** **]**	**Krupp et al. (2010) [** **34** **]**	**Shetty et al. (2021) [** **35** **]**	**Venturas and Umeh (2017) [** **36** **]**	**Wong (2011) [** **37** **]**	**Total no. of sources**
Awareness and knowledge of HCPs regarding HPV vaccination	∗	∗	∗	∗	∗	∗	∗	7
Attitude of HCPs towards HPV vaccination	∗	∗	∗	∗	∗	∗	∗	7
Perceived barriers to HPV vaccination	∗	∗	∗	∗	∗	∗	∗	7
Acceptance of HPV vaccination		∗	∗		∗	∗	∗	5

*Note:* The asterisks show the individual studies where the themes were derived.

The included studies used various qualitative methods; three conducted semistructured interviews [32, 34, 36], two used in‐depth interviews [33, 37] and one each employed key informant interviews [31] and focus group discussions [35]. Additionally, different qualitative analytical frameworks were applied, with three studies using thematic analysis [32, 33, 36], one study each employing content analysis [31], thematic content analysis [35], iterative analytic process [34] and grounded theory [37]. The differences in methods could be attributed to the research questions, the study′s context and the aim for understanding the problems under investigation.

Four main themes were identified in this review, which reflect HCPs′ knowledge, attitudes and barriers relating to HPV vaccination in LMICs. The themes were awareness and knowledge of HPV vaccination, attitude of HCPs towards HPV vaccination, perceived barriers and acceptance of HPV vaccination.

### 3.1. Awareness and Knowledge of HPV Vaccination

Across the studies reviewed, the findings demonstrated varied degrees of awareness and knowledge. Regarding high awareness and knowledge of HPV vaccination, two out of the seven studies demonstrated high awareness and knowledge about HPV and its associated vaccination. For example, in Nigeria, HCPs in tertiary health facilities had a high knowledge of HPV [31]. Similar evidence was reported among undergraduate healthcare students in India, in which there was a comprehensive understanding of HPV infection, including its risk factors and the benefits of vaccination [35].

‘Vaccination …between the ages of 9 and 26…after the age of 26, people still develop immunogenicity.’ (Family Physician 1, tertiary HF, Balogun and Omotade [31]).

‘Yeah [I have heard of the vaccine]. There are two types—Cervarix and Gardasil. Cervarix prevents against two strains and Gardasil prevents against four strains.’ (MBBS female, Shetty et al. [35]).

However, it was observed in studies conducted in India [33], Malaysia [37], Zimbabwe [32] and India [34] that HCPs showed little to low awareness and knowledge of HPV vaccination. Kataria et al. [33] reported that although physicians were generally aware of HPV vaccination, their understanding of specific details, such as the recommended dosing schedule and the full scope of its benefits, was limited. Additionally, physicians of different specialties had low knowledge of HPV and how it is linked with cancer of the cervix [34]. In Malaysia, Wong [37] reported that although HCPs recognized the risks associated with HPV, gaps in awareness and knowledge about the vaccine persisted. Despite the limited knowledge of HPV and vaccination, HCPs in Zimbabwe attributed a reduction in cervical cancer and genital warts to HPV vaccination [32].

‘The awareness is not there, many know vaccine is to prevent infectious diseases, but this is cancer. So for them to understand need a bit of time.’ (Malay female, family physician, 45, Wong, [37]).

‘I think anyone [physician] can recommend after knowing [about] the vaccine and they [adolescents] can take it from the gynaecologist.’ (General physician, Howrah (rural), Kataria et al. [33]).

‘…most doctors do not know [enough about] this vaccine. If they knew more they would push it through.’ (Paediatrician, Krupp et al. [34])

‘I think it is good…because it is aimed at reducing this disease! So maybe prevention is better than curing.’ (P7, Crann et al. [32])

### 3.2. Attitudes of HCPs towards HPV Vaccination

Evidence from five of the included studies [31–35] shows positive attitudes by HCPs regarding HPV vaccination. In Nigeria, HCPs in secondary health facilities were optimistic that recommending the HPV vaccine could enhance its uptake [31]. This aligns with the findings of Crann et al. [32] and Shetty et al. [35]. In addition to a sense of responsibility, Krupp et al. [34] reported that most participants in their study expressed broadly positive views about HPV vaccines, with an emphasis on the vaccine′s role in cancer prevention. This aligns with the findings of Crann et al. [32] and Shetty et al. [35]. HCPs believed in the efficacy of the vaccine and its role in reducing the burden of HPV‐related diseases [32, 35]. In India, most physicians believe discussions on HPV vaccination and cervical cancer with parents and adolescents were opportunistic [33].

‘I will recommend the [HPV] vaccine because cancer [of the] cervix is one thing the whole of Indian women are scared of… Prevention is always better than a cure…’ (Paediatrician, Krupp et al. [34])

‘When parents question too much, I leave it to them and do not push further. Will not force them. And it is also not like the government of India is recommending it.’ (Paediatrician, Kolkata (urban), Kataria et al. [33])

‘Most people I have recommended it to, they have taken it.’ (Family Physician 1, Tertiary HF, Balogun and Omotade [31])

urthermore, some HCPs exhibited negative attitudes towards HPV vaccination, and this was even realised in areas where some HCPs had information about the vaccine, leading to their reluctance to recommend the vaccine to the targeted groups. For instance, HCPs in a primary health centre in Nigeria and an urban health facility in India were not recommending the vaccine due to inadequate information about the vaccine [31, 33]. Additionally, Venturas and Umeh [36] reported misinformation regarding the HPV vaccine in Zambia, with HCPs expressing doubts about its importance and effectiveness. These skepticisms were often rooted in cultural beliefs, misconceptions about the vaccine or misinformation regarding the vaccine′s potential side effects.

‘What I have heard about the vaccine, some say that it has been developed to reduce the population, to reduce the fertility in a woman, an African woman.’ (Participant J, Venturas and Umeh [36])

‘If somebody is asking me at the age of 11 if I need to give the HPV vaccine, I would say no, because I am not very convinced myself that sexual debut starts that early here. I would probably say, “No, you do not need it at this age. You could wait.” I would be comfortable in giving around 16 years.’ (Paediatrician, Kolkata (urban), Kataria et al. [33]).

‘I do not recommend unless there is full knowledge of side effects and safety. If there is any accident or some intolerable side effect the public opinion can be very bad. We had some incidents with measles vaccinations; there was a furor about it [across] the whole country. Once it gets into news media, it has a negative effect on our other immunizations so I won′t recommend it unless I′m sure it is safety.’ (Paediatrician, Krupp et al. [34]).

### 3.3. Perceived Barriers to HPV Vaccination Among HCPs

Evidence from three of the included studies conducted in Zambia [36] and India [33, 35] revealed interpersonal, community‐level and systemic barriers.

### 3.4. Interpersonal Barriers

Studies conducted in Zimbabwe [32], Malaysia [37] and Zambia [36] found that some parents were hesitant to consent to the HPV vaccine due to concerns about its safety, side effects and perceived inappropriateness for their children. Krupp et al. [34] equally identified parental hesitancy as a major barrier to HPV vaccination, noting that many parents were unaware of the vaccine′s benefits or held misconceptions about its purpose. In certain cultural contexts, some parents feel uncomfortable discussing issues regarding sex, including sexually transmitted infections [34].

‘I feel they have not done much work on it for them to even start giving a vaccine. And this is why I was not comfortable for my daughter to have the vaccine.’ [Participant I, Venturas and Umeh [36])

‘These things are very personal, particularly in our country. People do not want to talk to their children about sex. It becomes very difficult so I think most of my patients will not respond well. Maybe when we are talking about menstrual hygiene we can be indirectly talking about some subjects. Talking about sexually transmitted infections is hard since most people won′t think their daughter could be sexually active. This is a problem for this vaccine. Most parents will not consider it, and I think it would be hard in a private practice to try to make people understand why they should take it.” (Obstetrician gynaecologist, Krupp et al. [34])

### 3.5. Community‐Level Barriers

The influence of culture and sociocultural values has an important effect on an individual′s attitude, regardless of their level of education or expertise. Culture significantly influences the perception of illness and the seeking of healthcare in LMICs. HCPs may encounter situations related to sociocultural beliefs, gendered norms and systems, such as patriarchy. Most LMICs embrace the patriarchal system, which allows individuals to freely dominate or influence decision‐making, including health‐related choices such as vaccination. For example, in many patriarchal societies, females often require the permission of male family members or older relatives to access healthcare services, including vaccination [36, 35]. Again, culture may subtly influence HCPs working in such geographic jurisdictions to express their cultural biases and gradually communicate or advise women on HPV vaccination.

‘The cultural background, that a woman should seek permission from her husband, whether she should take her daughter for the vaccine. So those are cultural issues that will always be there.’ (Participant K, Venturas and Umeh [36])

‘People here would judge you knowing that you are sexually active and that is why you will be taking the vaccine.” (MBBS, female, Shetty et al. [35])

‘…Many of these subjects are taboo, so it is hard for a doctor to talk about a vaccine that concerns sex especially if it is about an adolescent girl of nine or 10 years. It might be ok if it was just a cancer vaccine but not otherwise.’ (Obstetrician/gynaecologist, Krupp et al. [34])

### 3.6. Systemic Barriers

Economic and health system challenges emerged as critical barriers hampering HPV vaccination. The high cost of the HPV vaccine poses a significant burden, particularly in Zimbabwe [32], India [32, 34], Zambia [36] and Malaysia [37]. In these settings, both patients and healthcare facilities often struggle to afford the vaccine, leading to limited access and reduced uptake.

‘Other than monetary issues, I have no hesitancy because it is an expensive vaccine. Because after hearing the cost, some parents cannot afford it. That is a very sad part of it.’ (Paediatrician, Kolkata, (urban), Kataria et al. [33])

‘Because the price is too high, so sometimes they think that we are the one who mark up the price. So, in a way also sometimes we… that is why we are selective to which patient we talk to. That is why we do not talk to every patient about the vaccine.’ (Chinese male, gynaecologist, 49, Wong [37]).

‘People have money, but they do not want to spend. A vaccine like this has to be pocket‐friendly otherwise people will not take it. I treated a child with pneumonia. He almost died but his family was still hesitating to immunize [their] other children with pneumococcal vaccine. They said they will think about it and then left. They are optional vaccines, so people resist. I asked them “Is your child optional?” but they do not listen. This will be the biggest problem with the HPV vaccine. Unless it is part of the immunization schedule most people won′t take it even if I tell them they should.’ (Paediatrician, Krupp et al. [34]).

Additionally, communication barriers within the healthcare system are another major obstacle [32, 33]. Kataria et al. [33] identified that limited patient–physician time during consultations makes it difficult for HCPs to provide comprehensive information about the HPV vaccine and address patient concerns. The lack of time limits the ability of healthcare workers to engage in meaningful discussions with patients, reducing the chances of educating them about the importance of vaccination.

‘Time is important. You need 5 min to explain the full thing. They are seeing 10 patients and they are very quick [visits] and [I] do not have time [to explain this vaccine].’ (General physician, Kolkata (urban), Kataria et al. [33])

‘We have morning OPD, maybe 25 or 30 patients. It is difficult to talk in‐depth. We try to make sure of the main vaccinations. People know from the government schedule so it is not so hard, an optional vaccine, this is harder. If they have read and come to us to ask it is ok because they are already aware, then it is difficult. If they are not, it is hard to answer [questions] in a short time.’ (Paediatrician, Krupp et al., [34])

Also, information and safety concerns impact vaccine acceptance in India and Zimbabwe [32, 35]. Shetty et al. [35] noted that unreliable sources of information about the HPV vaccine contribute to confusion and fear. This misinformation creates a sense of uncertainty, which can discourage both HCPs and patients from pursuing vaccination. Moreover, safety concerns, particularly regarding potential side effects, further exacerbate these issues. It was identified in rural Zimbabwe that unreliable electricity and inadequate transportation systems were challenges to HPV vaccination [32].

‘I have taken two doses of vaccine so far, and I have not had any complaints or side effects.’ (Shetty et al. [35]).

‘It [would] be okay so long as the generators are working…We have some cooler boxes and refrigerators.’ (P6, Crann et al. [32])

### 3.7. Acceptance of HPV Vaccination by HCPs

Acceptance varied across studies conducted in India and Zimbabwe [32, 33, 35]. For instance, Shetty et al. [35] found that females exhibited a high level of acceptance, recognizing the importance of the vaccine in preventing cervical cancer and other HPV‐related diseases. However, low acceptance was also noted in Zambia [36] and Malaysia [37] which was due to parental hesitancy.

‘… I′ve already received my vaccination … but I think everyone should get vaccinated …’ (Female, MBBS student, Shetty et al. [35]).

‘I think it is a good idea…I do not think there will be anything bad about it. If there is, it will be one in a million…so I would rather go for it.’ (P10, Crann et al. [32]).

‘The females what they think about it, the others think that it is the wrong thing, they are scared, others they accept, but most of them they are scared. When the children tell their parents they want to give us this vaccine, most of the parents they were refusing saying “no us we do not know about this drug”, so they refuse.’ [Participant M (L: 19–22), Venturas and Umeh [36]).

‘Parents of children age 9 to 11 are not so open to the idea yet. They do not see their children need it, still in primary school. Majority recipients are around the age 15 years old. In terms of ethnicity, the Malays are less responsive. Among the Muslims, we have also got the very religious and the moderately religious. Those very religious type, they do not even want to listen, do not even want to know about the vaccine.’ (Malay male, paediatrician, 43, Wong [37])

## 4. Discussion

We conducted a systematic review of literature on HCPs′ knowledge, attitudes and barriers related to HPV vaccination in LMICs. The purpose of this review was to provide an understanding of the challenges HCPs face in promoting HPV vaccine uptake. Our findings revealed varying degrees of awareness and knowledge of HPV vaccination among HCPs, with some demonstrating a comprehensive understanding. This can facilitate effective patient education and vaccination efforts, as informed professionals are better equipped to promote the vaccine [39]. However, some HCPs demonstrated little knowledge of HPV vaccination across several studies. These gaps in understanding highlight the need for targeted educational interventions to enhance HCPs′ knowledge and ensure that they are fully equipped to educate patients effectively. Without such interventions, HCPs with low knowledge may inadvertently hinder HPV vaccination advocacy efforts and potentially hinder vaccine uptake. These findings emphasize the need for comprehensive training programmes to address these knowledge deficits and strengthen HPV vaccination initiatives in LMICs [40].

Furthermore, it was evident that some HCPs demonstrated positive attitudes towards HPV vaccination. This means that they generally understand the importance of the HPV vaccine and are motivated to advocate for it, contributing to positive vaccination practices within their communities. These practitioners may have a strong belief that it is their responsibility to recommend the vaccine to parents for use in adolescents and encourage them to receive it. In Nigeria, for instance, HCPs felt that it was essential to recommend the vaccine to young people [31]. This sense of duty underscores their commitment to safeguarding public health by increasing vaccination coverage. Similarly, in India HCPs had favourable attitudes towards the vaccine, with most believing in the importance of vaccinating adolescents to prevent HPV‐related cancers [33]. Female HCPs, in particular, demonstrated a positive attitude towards the vaccine [35]. This may be attributed to a greater awareness of the importance of preventing cervical cancer, a disease that disproportionately affects women. Nonetheless, negative attitudes, particularly stemming from a lack of trust in the vaccine′s safety or purpose, can significantly hinder vaccine promotion and uptake. This is because HCPs may not feel confident in encouraging patients to receive the vaccine. This negative perception may result in HCPs being less inclined to recommend the vaccine to their patients, potentially undermining vaccination efforts.

Our findings further suggest that HCPs face numerous barriers that hinder successful vaccine delivery and uptake. Notably, this review identified interpersonal, community‐level and systemic barriers. The interpersonal barriers reported by the HCPs include parents being hesitant as a result of misconceptions due to inadequate information about the vaccine. It was observed across some studies that some parents felt uncomfortable discussing issues related to sex with their children. An earlier study reported that HCPs often face difficulty interacting with parents, especially when discussing sensitive topics like HPV infection [34]. The stigma and cultural taboos surrounding sexual health make these conversations particularly challenging, preventing open dialogue about the vaccine and hindering its promotion. This could lead to missed vaccination opportunities [33, 35, 36]. Additionally, community‐level factors identified to be an obstacle to HPV vaccination were sociocultural in nature. It was observed that in some settings, the vaccine was viewed as inappropriate due to some deep‐rooted cultural beliefs surrounding sexuality and traditional gender roles. For instance, some females encounter difficulties in accessing health services, including HPV vaccination due to the need to require permission to access such services.

The systemic factors identified were the high cost of the vaccine and communication challenges within the healthcare system as these could potentially stall HPV vaccination uptake. The cost of the vaccine is a major factor hampering HPV vaccination uptake, especially in some LMICs where there are no national programmes to ensure effective vaccination for all eligible individuals. It is important that governments deliberately integrate HPV into national immunization programmes. Aside from the direct cost of the vaccine, the cost of travelling to healthcare facilities adds an additional financial burden, making it more difficult for them to access the vaccine. This issue is compounded by the lack of transportation infrastructure and unreliable electricity in some areas, which creates storage issues and further limits access to vaccination services [32]. This mostly affects facilities in rural settings. The potential effects of these challenges are the reduction in the uptake and efficacy of available vaccines. This result suggests the need for logistics to maintain the cold chain to ensure effective HPV vaccination.

This review further indicates that information and safety concerns also impact vaccine acceptance. It was evident that HCPs either had limited or no time with their patients and consequently were unable to have meaningful discussions on HPV vaccination. Shetty et al. [35] noted that unreliable sources of information about the HPV vaccine contribute to confusion and fear. This misinformation creates a sense of uncertainty, which can discourage both HCPs and patients from pursuing vaccination.

Despite challenges with HPV vaccination, its acceptance is critical to the elimination of HPV‐related cancers. HCPs who are open to and supportive of the vaccine are more likely to recommend it to patients, whereas those with low acceptance can create barriers to vaccine promotion. Across some of the studies reviewed, acceptance of HPV vaccination was high. This may be attributed to a better understanding of the vaccine′s benefits, particularly in communities where cervical cancer is a significant public health concern. Female HCPs, particularly, were more likely to advocate for vaccination, which underscores the positive influence of gender‐specific health concerns in shaping attitudes towards the vaccine [35]. Low acceptance, however, could be a key barrier, as HCPs are key influencers in vaccine uptake. Consequently, their reluctance to endorse the vaccine can lead to missed opportunities for vaccination and lower overall vaccination rates.

It is worth mentioning that four of the reviewed studies were published before the Coronavirus 2019 (COVID‐19) pandemic [33, 37, 31, 35], whereas three were after [32, 34, 36]. A critical reflection of the findings indicates that awareness of HPV vaccination appeared lower before COVID‐19 compared with after [32, 34, 36]. However, attitudes towards HPV vaccination showed no clear difference between pre‐ and post‐COVID‐19 studies, with both positive and negative attitudes documented in each period. Although barriers to HPV vaccination persist post‐COVID‐19, vaccine acceptance has improved compared with the prepandemic era. It has been documented that the COVID‐19 pandemic led to a lack of access to HPV vaccines in LMICs, but due to numerous strategies to increase uptake, public confidence in the vaccine is gradually increasing [41].

A critical analysis of the findings suggests that although the study covered different cadre of HCPs, physicians were the predominant group. It is unclear whether any specific population or specialty of practice had greater knowledge or more positive attitudes. For instance, within the same study, some doctors and nurses demonstrated high knowledge and positive attitudes, whereas others with similar qualifications showed limited knowledge. This suggests that differences in knowledge and attitudes are more likely related to the geographical location of the study rather than the population or specialty. Therefore, it was not conclusive whether a particular subgroup had more knowledge and positive attitudes. Across the various cadre of HCPs, there seems to be consistent gaps even in regions where some have demonstrated knowledge and positive attitudes towards vaccination. Therefore, there should be deliberate efforts to build the capacity of HCPs, including students to improve HPV vaccination efforts in LMICs.

A typical capacity building intervention to improve HCPs′ attitudes and knowledge of HPV vaccination in LMICs could adopt the following framework for implementation inspired by the six steps in quality intervention development (6SQuID) [42, 43]. Firstly, an assessment of needs and understanding of the problem could be conducted with HCPs to gauge current knowledge, attitudes and practices around HPV vaccination, using for instance this current review as a starting point but contextualising the findings to examine HCPs′ familiarity with national immunization guidelines and policies, as well as examining cultural, logistical and policy barriers affecting HPV vaccine uptake. The assessment of needs could also involve resource mapping by identifying available training materials and digital tools that could be employed. The next stage within an implementation framework involves assessing what needs to change to address the problem. Knowing this could help with goal setting and prioritisation. The goal could be to strengthen technical knowledge and policy understanding among HCPs to improve HPV vaccination rates. Once a clear goal has been set, it is important to clearly design and plan the strategies to be implemented. Using a logic model could help to diagrammatically articulate the change process and the resources that will be required to bring about change.

With clear plans in place, implementation of activities could then ensue, mobilising relevant resources (e.g. NGO partners) or existing support systems (e.g. government health programmes). Capacity building activities could include short, focused learning sessions (in‐person or virtual) covering HPV facts, safety and policy updates; interactive workshops focusing on communication techniques for vaccine advocacy [44]; brief refresher sessions on national vaccine policy and guidelines for HCPs; and a train‐the‐trainer approach, where local HCPs could be equipped to cascade knowledge. Dissemination of accessible policy briefs and infographics will be useful at this stage. To ensure these activities are being implemented as intended, regular supervisory visits or virtual check‐ins to reinforce learning and troubleshoot will be required.

Monitoring and evaluation should be instituted to ensure ongoing improvement and sustainability of the capacity building intervention. This could be undertaken by measuring knowledge and attitudes before and after the intervention is implemented. Assessment of HCPs′ communication with patients regarding HPV vaccination could also be examined by practice observation. Long‐term improvements could be assessed by monitoring HPV vaccine uptake in the community and nationally. Finally, to ensure sustainability, HPV education content could be embedded in ongoing continuing professional education programmes and courses.

## 5. Strengths and Limitations

A key strength of this systematic review is that it addresses a significant gap in the literature by being the first to explore HCPs′ knowledge, attitudes and barriers relating to HPV vaccination in LMICs. The protocol for this systematic review was strictly followed and data extraction was conducted in duplicate to minimise the risk of bias and ensure reliability. However, there were limitations. The review was restricted to publications in English based on the eligibility criteria, which may have excluded potentially relevant studies in other languages. The included studies employed varied qualitative data collection methods and analytical frameworks, which could affect the consistency and interpretation of the findings. Despite these limitations, the review offers invaluable insights into the factors that influence HCPs′ roles in promoting HPV vaccination in LMICs. Additionally, the small number of studies included in this review could impact the conclusions and transferability of the findings. This, however, shows that the phenomenon is not widely studied, and there are limited published studies on the topic. However, a rigorous process was followed, and the seven studies met the criteria for inclusion. This is an indication of the limited research evidence in this topic area within LMICs. Therefore, the generalizability of the findings to all LMICs should be done with caution. Future reviews on this topic should focus on quantitative or mixed methods studies.

## 6. Conclusions

This review has highlighted critical areas for consideration regarding HPV vaccination among HCPs in LMICs. Across the included studies, there was evidence of significant gaps in knowledge and variability in attitudes towards HPV vaccination. Furthermore, significant barriers, ranging from sociocultural and economic challenges to systemic and parental hesitancy, were identified as factors that could undermine HPV vaccination efforts in LMICs.

To address these issues, comprehensive interventions to equip HCPs with the accurate and relevant information and resources about HPV vaccination are urgently needed. Such interventions could enhance their knowledge, improve their attitudes and potentially eliminate or minimise existing barriers. Additionally, strengthening health systems to address systemic barriers is crucial to achieving the WHO′s goals for HPV vaccination in LMICs.

## Ethics Statement

The authors have nothing to report.

## Consent

The authors have nothing to report.

## Disclosure

All authors reviewed, edited and approved the final manuscript.

## Conflicts of Interest

The authors declare no conflicts of interest.

## Author Contributions

Conceptualisation: Nancy Innocentia Ebu Enyan, Sebastian Ken‐Amoah, Patrick Kafui Akakpo, Dorcas Obiri‐Yeboah and Lawrence Doi. Methodology: Nancy Innocentia Ebu Enyan, Mengying Zhang, Sebastian Ken‐Amoah, Michelle King‐Okoye, Patrick Kafui Akakpo, Dorcas Obiri‐Yeboah and Lawrence Doi. Software: Mengying Zhang and Michelle King‐Okoye. Data curation: Mengying Zhang and Dorothy Addo‐Mensah. Investigation: Nancy Innocentia Ebu Enyan, Mengying Zhang, Michelle King‐Okoye, Joyce Agyeiwaa, Richard Sarfo‐Walters, Dorothy Addo‐Mensah, Dorcas Obiri‐Yeboah and Lawrence Doi. Validation: Nancy Innocentia Ebu Enyan, Mengying Zhang, Sebastian Ken‐Amoah, Joyce Agyeiwaa, Richard Sarfo‐Walters, Dorothy Addo‐Mensah, Dorcas Obiri‐Yeboah and Lawrence Doi. Formal analysis: Nancy Innocentia Ebu Enyan, Michelle King‐Okoye, Joyce Agyeiwaa, Richard Sarfo‐Walters, Dorothy Addo‐Mensah, Dorcas Obiri‐Yeboah and Lawrence Doi. Supervision: Nancy Innocentia Ebu Enyan, Sebastian Ken‐Amoah, Patrick Kafui Akakpo, Dorcas Obiri‐Yeboah and Lawrence Doi. Project administration: Nancy Innocentia Ebu Enyan, Dorcas Obiri‐Yeboah and Lawrence Doi. Resources: Nancy Innocentia Ebu Enyan, Dorcas Obiri‐Yeboah and Lawrence Doi. Writing draft: All authors contributed to the draft manuscript and writing—review and editing.

## Funding

No funding was received for this manuscript.

## Supporting information


**Supporting Information 1.** Additional supporting information can be found online in the Supporting Information section. File S1: Critical appraisal of included studies.

## Data Availability

Data sharing not applicable as no datasets were generated and/or analysed for this study.
