# Supplementary material for: Acceptance of HPV Vaccination: A Systematic Review of Knowledge, Attitudes and Barriers Among Healthcare Practitioners in Low‐ and Middle‐Income Countries
Source: Biomed Res Int. 2026 Jan 16;2026:9294978. doi: 10.1155/bmri/9294978 (PMC12809713; doi:10.1155/bmri/9294978)
Supplement: Supplementary file 1 — Supporting Information 1. Additional supporting information can be found online in the Supporting Information section. File S1: Critical appraisal of included studies. [file BMRI-2026-9294978-s001.docx]

| **Author/s** | **Is there congruity between the stated philosophical perspective and the research methodology?** | **Is there congruity between the research methodology and the research question or objectives?** | **Is there congruity between the research methodology and the methods used to collect data?** | **Is there congruity between the research methodology and the representation and analysis of data?** | **Is there congruity between the research methodology and the interpretation of results?** | **Is there a statement locating the researcher culturally or theoretically?** | **Is the influence of the researcher on the research, and vice- versa, addressed?** | **Are participants, and their voices, adequately represented?** | **Is the research ethical according to current criteria or, for recent studies, and is there evidence of ethical approval by an appropriate body?** | **Do the conclusions drawn in the research report flow from the analysis, or interpretation, of the data?** | **Total score (YES=1, NO=0, NOT CLEAR=0)** |
| --- | --- | --- | --- | --- | --- | --- | --- | --- | --- | --- | --- |
| Balogun & Omotade (2022)  **“Facilitators and barriers…”** | YES | YES | YES | YES | YES | YES | YES | YES | YES | YES | 10 |
| Crann et al. (2016) “**Healthcare providers’ perspectives..”** | NOT CLEAR | YES | YES | YES | YES | NO | NO | YES | YES | YES | 7 |
| Kataria et al. (2022)  **“Awareness, perceptions, and choices…”** | YES | YES | .YES | YES | YES | NO | NO | YES | YES | YES | 8 |
| Krupp et al. (2010) **Factors Associated With.”** | YES | YES | YES | YES | YES | .NO | NO | YES | YES | YES | 8 |
| Shetty et al. (2021)  **“An exploratory study …”** | YES | YES | .YES | YES | YES | YES | NO | YES | YES | YES | 9 |
| Venturas & Umeh (2017) **“Health professional feedback..”** | YES | YES | .YES | YES | YES | .YES | NO | YES | YES | YES | 9 |
| Wong (2010), “**Issues Surrounding HPV Vaccine…”** | NOT CLEAR | YES | YES | YES | YES | NO | .NO | YES | YES | YES | 7 |
